# Covid-19 and Kawasaki syndrome

**DOI:** 10.1017/S1047951120001894

**Published:** 2020-06-18

**Authors:** Viroj Wiwanitkit

**Affiliations:** Dr DY Patil University, Pune, India

Dear Editor,

I would like to discuss on “Covid-19 and Kawasaki Syndrome: Should We Really Be Surprised?^1^” Loomba et al. concluded that it was no surprising that there might be an association between the novel coronavirus infection and Kawasaki syndrome.^1^ Indeed, it is no doubt for possible association since many viruses can induce Kawasaki syndrome. Nevertheless, the surprising issue should be the discrepancies in clinical presentation in different settings. It is interesting that there is no report on Kawasaki-like syndrome in COVID-19 patients during its first phase of pandemic in East Asia and Southeast Asia. The problem was first noted when the disease came to the West. The underlying genetic factor might be a factor that cause more common Kawasaki-like syndrome in non-Asian population. Indeed, the intravenous immunoglobulin unresponsiveness in non-Asian patients with Kawasaki disease is reported and the difference varied among different ethnic groups.^2^

## References

[1] Loomba RS , Villarreal E , Flores S Covid-19 and Kawasaki syndrome: should we really be surprised? Cardiol Young. 2020 May 15. [Epub ahead of print]. doi: 10.1017/S1047951120001432 PMC7322149 32412400

[2] Piram M , Darce Bello M , Tellier S , Di Filippo S , Boralevi F , Madhi F , Meinzer U , Cimaz R , Piedvache C , Koné-Paut I Defining the risk of first intravenous immunoglobulin unresponsiveness in non-Asian patients with Kawasaki disease. Sci Rep. 2020; 10: 3125. 32080307 10.1038/s41598-020-59972-7 PMC7033244

